# ECONOMIC IMPACT OF DEMENTIA ON AFRICAN AMERICAN AND LATINO ADULTS: A GROWING CHALLENGE

**DOI:** 10.1093/geroni/igae098.1154

**Published:** 2024-12-31

**Authors:** Stipica Mudrazija, Maria Aranda, Darrell Gaskin, Patrick Richard, Stephanie Monroe

**Affiliations:** University of Washington, Seattle, Washington, United States; University of Southern California, Los Angeles, California, United States; Johns Hopkins University, Baltimore, Maryland, United States; Uniformed Services University, Bethesda, Maryland, United States; UsAgainstAlzheimer’s, Washington, District of Columbia, United States

## Abstract

In this study, we estimate the economic impact of dementia today and through 2060 associated with a) lost wages of persons living with dementia as well as their caregivers, b) excess medical care spending for persons living with dementia, c) value of unpaid care, and d) the value of wider economic costs (lost productivity, lost tax revenue), for African American and Latino adults living in the US and their caregivers. In 2020, the total economic impact of dementia for African American and Latino adults reached almost $94 billion, including over $62 billion (66%) in unpaid care and over $31 billion (34%) in various costs for older adults, their caregivers, and others. Latino and African American adults accounted for 60 and 40 percent of the costs, respectively. By 2060, the economic impact of dementia for African American and Latino adults will increase to $1.4 trillion (in 2060 dollars). The share of the total attributable to unpaid care will decline from 67 percent to 60 percent, while the share of various costs will increase, and most notably forgone wages (from 21% to 27%). The shift in the cost distribution is due mainly to shifts in the caregiving population’s age distribution and educational attainment level. Efforts aimed at keeping working-age caregivers attached to the labor force will become increasingly important to limit the fast growth in the value of work-related costs of caregiving.

